# The MDENet education platform: zero-install directed activities for learning MDE

**DOI:** 10.1007/s10270-025-01292-3

**Published:** 2025-06-02

**Authors:** Steffen Zschaler, Will Barnett, Artur Boronat, Antonio Garcia-Dominguez, Dimitris Kolovos

**Affiliations:** 1 Department of Informatics, King’s College London, London, UK; 2 School of Computing and Mathematical Sciences, University of Leicester, Leicester, UK; 3 Department of Computer Science, University of York, York, UK

**Keywords:** MDE, Education, Online, No installation, Playground

## Abstract

Setting up and configuring model-driven engineering (MDE) tools is not straightforward because the MDE tooling landscape is highly fragmented and because many MDE tools are research prototypes with limited documentation. This creates significant accidental complexity for learners of MDE, who have to overcome installation and configuration hurdles before they can even begin to focus on the core MDE concepts they should be learning. This is further complicated by the complexity of modern MDE tools, which can overwhelm new learners, making it difficult for them to work out what they should do next to achieve a given goal. To address these challenges, we have developed a web-based playground platform that enables learners to engage with MDE learning activities without the need to install anything. The playground metaphor allows teachers to expose only those functionalities directly required for the completion of a particular learning activity. We present the general architecture of the platform, our approach to the declarative integration of new MDE tools, and the way in which teachers can flexibly and declaratively define new MDE learning activities. We have used our platform in a range of different contexts, from live tutorials and 10-week university courses, to developing documentation webpages for MDE tools. We describe examples of such uses, showcasing the flexible configurability of the platform for different types of activities and contexts.

## Introduction

Model-driven engineering (MDE) [7] is a paradigm where models play a central role in the development of a software system. Over the last couple of decades, MDE has been an area of active research with advancements in techniques and tools, and success stories in the real world [41]. In terms of education, there is a consensus that MDE is a complex subject to teach [14, 27].

A particular challenge comes from the complexity and availability of suitable tools [8, 12, 14]. We focus on two challenges in particular:

1. *MDE tools are difficult to install and configure correctly.* Most MDE tools depend on a rich ecosystem of other tools and frameworks, all of which need to come together in the right versions and configurations for a given tool to work. Installing multiple tools can easily lead to conflicting demands for different versions of the same underlying tool or framework. Tools are typically implemented in Java and often as part of the Eclipse ecosystem. As a result, learners of MDE first have to overcome a significant hurdle in getting to a workable MDE tool installation on their computer before they can even begin to learn MDE concepts and techniques. This challenge has also previously been identified in surveys of learners of modelling in UML [1].
2. *MDE tools are too powerful for learners.* Even when a learner has successfully installed the MDE tools required for a particular course, they can easily become overwhelmed by the complexity of the tools themselves [38, 39]. Most MDE tools are integrated into an IDE, which typically provides other capabilities, too. As a result, there are usually hundreds of menu options and tool bar buttons to choose from. Picking the right one for a given task quickly becomes challenging for novice MDE users. As a result, learners of MDE have to first learn which functionalities are relevant before they can focus on learning MDE concepts and techniques. Note that, in the context of teaching and learning programming, this challenge has also been recognised and has been the foundation of the development of bespoke education-focused tools [28, 29, 35]

These challenges create *accidental complexity* [25] for learners of MDE. We want learners to encounter difficulties, but these should be *desirable difficulties* [3] that enhance their learning, such as guided practical engagement with the relevant concepts. Ideally, learners would be able to focus on the MDE concepts and techniques they are trying to understand, rather than first having to overcome several accidental challenges.

There are good reasons for the added complexity *for production MDE tools.* Tools need to flexibly support a broad range of use cases and functionalities. However, much of this is not relevant for learners of MDE. We argue, therefore, that there is a need for MDE tools specifically for the purpose of learning MDE.

**Fig. 1:**
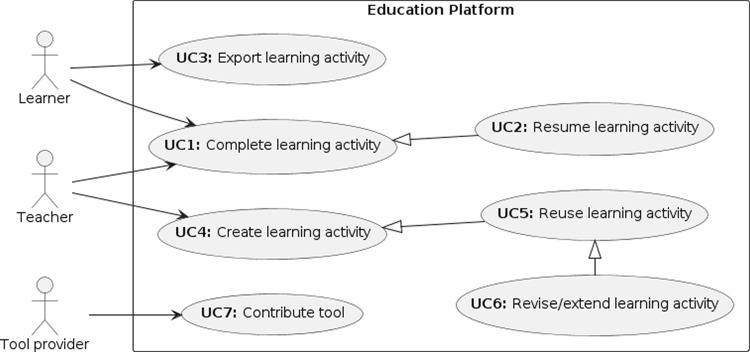
Stakeholders and key usage scenarios for the EP.

A recent workshop at MODELS 2023 identified a broad range of requirements for such modelling tools for teaching [27]. Here, we focus on a subset of these requirements. More specifically, we aim to address the following requirements:

1. Learners should be able to undertake MDE learning activities without the need to install and configure MDE tools.
2. Learners should be able to engage in different types of activities, such as typical model management activities or the creation of new languages using language workbenches.
3. Learners should be able to transition their MDE learning activities easily to a real-world tool environment.
4. Teachers should be able to easily and collaboratively define new learning activities.
5. Teachers should be able to control and constrain learners’ possible interactions with the MDE tools so that they can guide learners and avoid overwhelming them with complexity.
6. Teachers should be able to flexibly combine a range of MDE tools.
7. Tool providers should be able to easily contribute a new MDE tool for use in learning activities.

We address these requirements by providing an online playground environment for MDE learning activities—the MDENet Education Platform (EP in the rest of the paper). The web-based nature of the playground means there is no need to install anything beyond a basic web browser. The playground metaphor means that learners will only be exposed to a minimal interface focused on the files and functions required for a given learning activity. We provide a declarative language for flexibly defining learning activities. Learning activities are packaged as GitHub repositories, enabling teacher–teacher collaboration as well as providing learners with the ability to undertake the activities directly in standard IDEs if desired. Modelling tools for teaching should address requirements beyond the list above [27]. For example, such tools should provide support for automated assessment and rich feedback to learners. We do not currently address these further requirements in the EP. Note that the EP uses GitHub repositories to store assignments. This allows using GitHub Actions for auto-grading—for example by using the popular GitHub Classroom^1^ service.

This paper extends a paper presented at the MODELS Educators Symposium [2]. We extend that paper by giving an updated account of the EP’s architecture and design, as well as describing three case studies of how the EP has been used in different learning contexts and with different tools included in the learning activities. The EP already supports a breadth of MDE tools, including Epsilon, Emfatic, OCL, YAMTL, and Xtext, covering a spectrum of different installation requirements and user interactions, and the case studies we present showcase how some of these are integrated into learning activities.

The remainder of the paper is structured as follows: We introduce the three types of stakeholders and their respective use cases in Sect. 2. Section 3 uses a running example to illustrate an overview of the design of the EP. Section 4 describes three case studies of where the platform has been used, demonstrating different aspects of how we address the requirements above. Finally, after a brief discussion of related work in Sect. 5, Sect. 6 summarises the paper, indicates some future work, and provides information on how to get involved.

## Key platform users

Three types of key stakeholders are involved in teaching modelling and model-driven engineering (cf. Fig. 1):

1. *Learners* access the platform to complete learning activities created by a Teacher.
2. *Teachers* create lessons to deliver to their learners as activities on the platform, and they make available the activity files from a location accessible to their learners.
3. *Tool providers* create platform services for their existing tools. Activities use these services to perform MDE functions, such as model-to-model transformations, model validations, etc.

We next describe typical use cases for each of the three key stakeholders.

### Learners

Learners are interested in working through learning activities in order to improve their practical and conceptual understanding of MDE. Broadly, they are therefore interested to **complete learning activities (UC1)**. To do so, they access the learning activity via their teaching organisation’s Virtual Learning Environment (VLE) and work through the activity in a guided fashion (R1). Teachers may get involved in this – for example, by providing feedback or assessment of the learner’s work.

Learners may not complete an activity in a single sitting. They, therefore, need to be able to save their progress and **resume the learning activity (UC2)** at a later stage.

Finally, learners will eventually want to be able to learn about using real-world MDE tools, transitioning away from the “safe space” of the EP (R3). To support this step, learners need to be able to **export the learning activity (UC3)** in a way that makes it accessible to standard tools – for example by extracting a suitably formatted ZIP file or repository.

### Teachers

Teachers **create learning activities (UC4)** (R4). They may do so from scratch, and in this case are interested in being able to succinctly describe their learning activity, incorporating the most appropriate set of MDE tools (R6), including language workbenches (R2), and then store the learning activity so they can use it again and again in their teaching. In creating learning activities, teachers want to be able to constrain what learners can do, to ensure learners focus on the task at hand (R5)

To increase efficiency, teachers are also interested in **reusing existing learning activities (UC5).** To this end, learning activities should be stored in ways that can be easily shared with and accessed by other teachers. Of course, direct reuse of a learning activity *as is* may not always be appropriate. In those situations, teachers may wish to **revise or extend the learning activity (UC6).**

### Tool providers

A large community of researchers and practitioners develop MDE tools for a wide range of model-management and language engineering tasks. Tool providers are interested in making their tools accessible to learners of MDE (R7). To **contribute a tool (UC7)** to the EP, a tool provider needs to identify the key functionality and features that a learner needs to be able to access. They need to be able to package these functionalities in individually accessible parts, so that they can be appropriately combined in learning activities of different complexity levels.

## Architecture and design of the EP

The EP builds on the Epsilon Playground [33] but generalises the architecture to allow the declarative description of learning activities and the flexible integration of a wide range of MDE tools. The EP also integrates with GitHub to provide a way for learners to save their work and easily transition to the use of real-world MDE tools and environments.

In this section, we give an overview of the key components of the EP. First, we introduce an example learning activity, which we will use throughout the further explanations. We then start with a general overview of the architecture. Next, we describe how activities are defined and executed by the EP. We then describe how tools can be integrated with the EP, and the implicit model-type conversion provided by the EP to make tool integration easier. Finally, we briefly touch on support available for teachers and tool providers to make it easier to work with the EP.

### Running example

To make the description of the design, implementation, and use of the EP more concrete, we introduce a running example, to which we will refer as required. We reuse an example from the Epsilon Playground [33] focusing on the validation language EVL [31], which we have ported to the EP.^2^ The EP is available on GitHub.^3^ The example can be directly accessed on the publicly hosted version of the platform.^4^

**Fig. 2:**
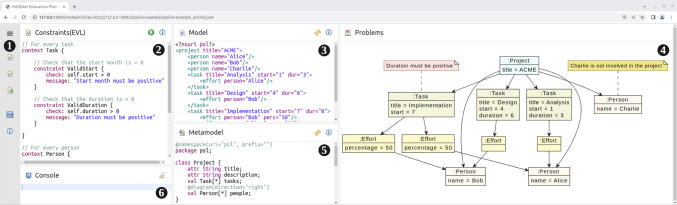
The Epsilon EVL example in the EP. Numbered circles indicate different parts referenced from the text.

Figure 2 shows the interface that learners see when completing the activity. In addition to a menu area ( ) on the left, there are five panels:

The contents of panel  are the constraints to check against the model  and its meta-model . Panel  displays the result of evaluating the constraints for the model. The console  shows error messages.

Learners edit constraints in Panel  and then click on the run button . This triggers the evaluation of the constraints the learner has provided. If there are errors in the constraint definition (for example, errors in the EVL syntax), these are reported in the console panel . Otherwise, the platform presents an annotated version of the model in Panel . In Fig. 2, the constraints were successfully evaluated, and two violations were identified (one task is missing a duration specification and “Charlie” is a person who is not involved in any project task).

### Platform architecture

**Fig. 3:**
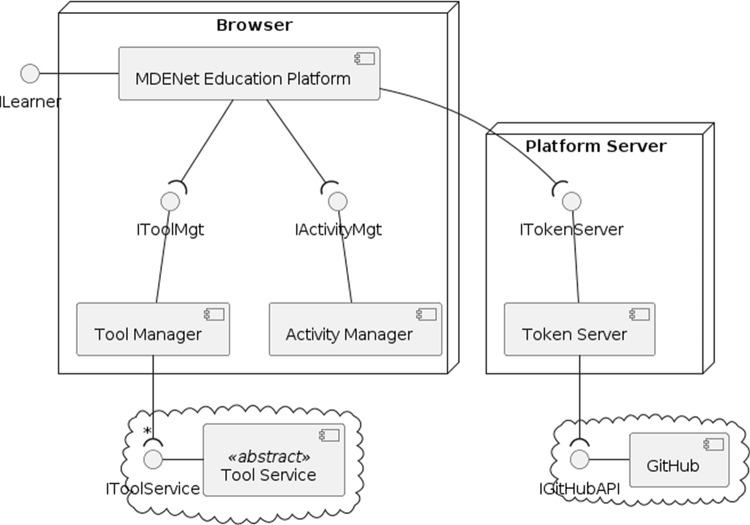
High-level architecture of the EP.

The EP is a single-page web application, with most of the functionality running directly in the learner’s browser. Figure 3 gives a high-level overview of the key components of the EP. The Platform Server provides the HTML and JavaScript to be executed in the learner’s browser. It also runs the Token Server, which provides authentication services for access to GitHub repositories (see below).

Three key components run in the learner’s browser:

1. The MDENet Education Platform provides the main entry point. It is responsible for reading activity specifications and activity files and setting up the user interface.
2. The Activity Manager is responsible for parsing and validating activity descriptions (see Sect. 3.3), enabling the EP to configure the appropriate user interface.
3. The Tool Manager keeps track of the tool services in use by the current learning activity. Tool Services implement wrappers around MDE tools to make them accessible to the EP (see Sect. 3.4). They are implemented (and typically hosted) by tool providers.

We implement a simple token server [15] to manage GitHub OAuth authentication tokens, which works together with a GitHub App^5^ and enables read and write access to the repository underlying a learning activity (assuming this is compatible with the learner’s access rights on GitHub). This means learners can easily save the current state of work as a commit to the underlying repository; the EP supports this directly through a “Save” button in the left-hand menu. Learners can then resume the activity at a later time (**UC2**).

### Defining activities

Learning activities are stored in GitHub repositories. Two types of files have to be provided:

1. A YAML [17] or JSON [20] file declaratively describing the configuration of the EP for the learning activity.
2. Any other files required for the learning activity—for example, models, language grammars, meta-models etc.

Learning activities are provided as a complete repository. This means teachers can include arbitrary files and folder structures beyond the files directly required for the learning activity. As a result, learners can transition to using regular IDEs and MDE tools if the repository contents have been set up so that it works directly with regular tools, and the learning activity definition only picks out those files directly required for the task at hand (R3). Learners can then checkout the repository to their computer outside of the EP to access it through regular tools and can even go back and forth between both modes of access at will.

#### Activity configuration

A learning activity is presented to the learner as a single web page with a collection of panels (  –  in Fig. 2). Teachers provide activity-configuration files to define the panels and functionalities available to the learner. An activity-configuration file may define multiple activities; these are shown as separate links in the left-hand side menu of the EP. In our running example, this menu can be seen in  in Fig. 2 and contains 3 activities, one of which is currently shown in the main platform space.

**Fig. 4:**
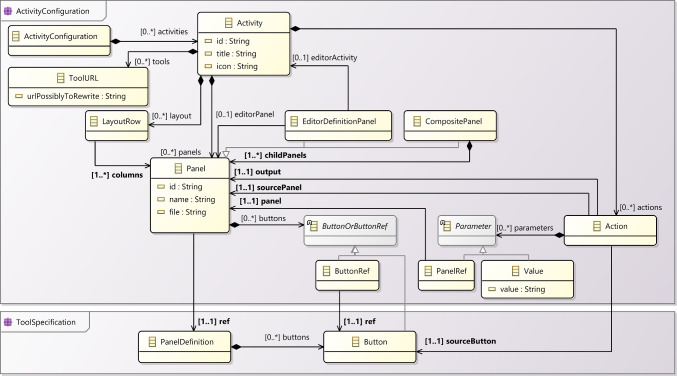
Meta-model of the activity-specification language.

To describe the set of learning activities available (**UC4**), a teacher uses a domain-specific language (R4), currently encoded as a JSON schema [26] (and, thus, also accessible via YAML [17]). We provide a graphical overview of the abstract syntax of the activity-specification language in meta-model notation in Fig. 4.^6^

In the following, we introduce the language step by step by walking through an example using the YAML notation.

Two kinds of common attributes used throughout the activity-configuration files are id and name (sometimes title). An id uniquely identifies the object that it is an attribute of and can be used in cross-references. A name or title is the text to display in user interfaces for the object.

Listing 1 shows the start of the top-level structure of the activity-configuration file for our running example (showing only one of the three defined activities). We have already described the id and title attributes. The icon attribute specifies the name of an icon defined in the images css static resource file for the icon in the activity menu (see  in Fig. 2).
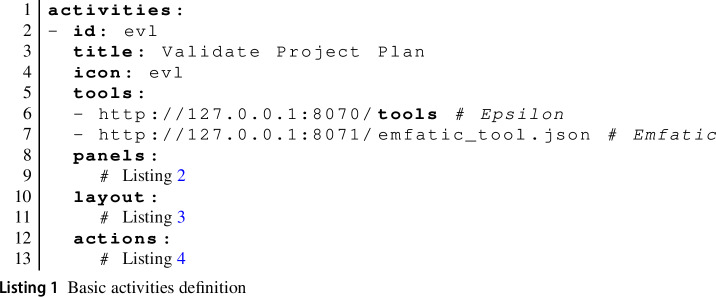


The tools array provides a list of URLs of the tool services for the MDE tools used in the activity. We support limited rewriting of these URLs. Specifically, a teacher can use the form {{ID-<panel-id>}} (where <panel-id> is the identifier of a panel in the current activity) which will be replaced by a URL for a tool service that has been generated as part of the completion of another activity. We will discuss this in more detail when we discuss language-workbench activities in Sect. 3.3.2. A complete example can be found in Sect. 4.2.3 on Page 26.

Tools can be deployed in arbitrary locations, which is why the tools array accepts fully specified URLs to identify the location of any tools. In Listing 1, the URLs we show are those used when hosting a local version of the EP, including the Epsilon tools and support for Emfatic [16]. For tools hosted with the platform, the EP is able to rewrite URLs using the notation {{BASE-URL}}:<port> followed by the name of a JSON/YAML file with the tool description. This is translated (based on a registry of port numbers) into appropriate paths at the base URL where the EP has been deployed. The two tool URLs from Listing 1 can then also be written as 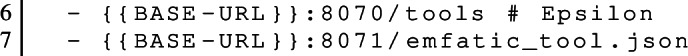 This notation will work regardless of whether the activity is executed on the publicly hosted EP, on a developer’s personal PC, or on a different server.

An activity definition further includes the definition of panels, their visual layout, and actions available to the learner. We will first consider the definition of panels and layouts, which specify what the learner can see in the browser.

An activity can define multiple panels. Each panel definition has id and name attributes as discussed above; the name is shown as the title bar of the panel on the platform. The ref attribute is used to identify the type of panel. Each tool can contribute new panel types, specifying syntax highlighting rules for text panels, panel icons, and buttons available by default. In addition, the EP defines some stock panel types; in particular, the console and composite panel types are defined directly by the EP. The ref attribute is also used to identify the type of the panel’s contents. This will become important when we discuss how the EP automatically transforms between types of contents when invoking functionality from different tools (cf. Section 3.5).

The file attribute is used to identify the file to be shown in the panel. This can be an arbitrary URL, but is normally a path relative to the path of the activity-configuration file in the containing GitHub repository. If so, the EP will commit any changes to the panel contents back to that file when the learner chooses to save their work (**UC2**). Panels, thus, allow teachers to focus learners’ attention on just the files they require for the current activity (R5).

Listing 2 shows the definition of the EVL panel for our running example. This assumes that the repository contains a file called psl.evl in the same folder as the activity-configuration file. The contents of that file will be shown in the panel and any changes will be committed back to that file when the learner chooses to save. The panel makes reference to the evl panel type, which is provided by the Epsilon tool previously referenced by its URL.
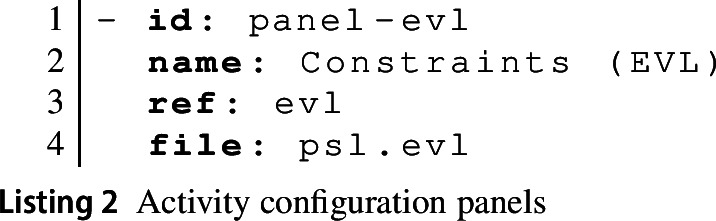


Two types of panels are worth discussing separately:

1. *Editor definition panels.* A key type of learning activity is about how to define new modelling languages and tools. This requires learners to go through a two-stage process where they first define a language and then try it out. The EP provides some special notation to couple these stages and we will discuss these in detail in Sect. 3.3.2 when discussing “language-workbench activities” in general.
2. *Composite panels.* At times, different perspectives on the same artefact are useful, but there may not be enough space on the screen to show them all simultaneously. For this purpose, the EP provides the option of defining composite panels, which contain other panels themselves. This is done by setting the ref attribute to composite. Special “toggle” buttons can be defined as part of the composite panel to allow learners to show and hide individual sub-panels. Composite panels can, in principle, be nested arbitrarily deeply. In practice, we have not yet found the need to define more than one level of nesting.

Panels can define buttons that the learner can use to trigger specific actions. Tool providers typically define default buttons for the panel types they contribute. These buttons can be overridden by teachers by providing a separate array of buttons in a panel specification (R5). Each element in such an array can either be a reference to one of the default buttons defined by the tool provider or a full separate button definition. We will describe button definitions in more detail in Sect. 3.4.

Once panels have been defined, the teacher needs to specify how these will be displayed. Note that it is possible to define panels that will not be shown. This can be a useful way of loading files required for certain functions without exposing the learner to the additional complexity. We will see examples of this in some of the case studies described in Sect. 4. Panels are shown by including them in the layout two-dimensional array. This has one element for each row of panels. Where arrays are of uneven lengths, panels will be resized to cover multiple rows automatically. Listing 3 shows the layout definition for our running example. Note how the panel-problems panel (Panel  in Fig. 2) is automatically expanded to fill both rows.
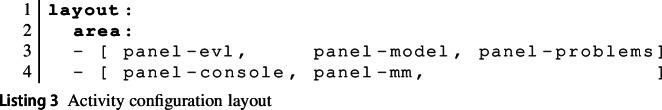


The final component of an activity definition is the definition of actions. These are used to define what happens when a learner clicks on a button on one of the panels. There are two parts to this:

1. Tool providers define web-based API endpoints (called functions) through which specific tool functionality is exposed. These have a name and a set of formal parameters. Buttons are associated with functions in the button definition. We discuss these in more detail in Sect. 3.4.
2. For a specific learning activity, an action specifies which panels provide the values for the various parameters (alternatively, values can be provided directly) and which panel will be used to display the output from the tool function. An action can also reference a separate console which can be used to display stdout/stderr output to the learner in addition to the actual function result. Listing 4 shows an example action definition for our running example. This defines what happens when the learner clicks on the action-button button in the panel-evl panel (the button with  on it). The tool provider has already defined this button to invoke a tool function that evaluates the EVL code over a model and meta-model. Here, we define where the EVL code (program) comes from (panel panel-evl) and what the model (panel-model) and meta-model (panel-mm) are for which the constraints are to be evaluated. Finally, we state that the result of the function invocation should be shown to the learner in panel panel-problems.

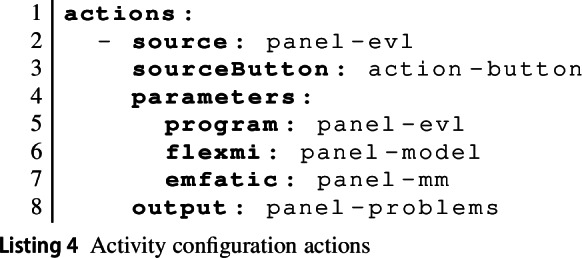


#### Language-workbench activities

A key aspect of MDE is the development of new, typically domain-specific, modelling languages. Therefore, the EP needs to be able to support learning activities that require learners to develop their own modelling languages (R2). Such activities take two steps:

1. Learners *create* a description of their modelling language. This may be a meta-model, a grammar, a specification of the graphical syntax, a code generator template, validation code, etc.
2. Learners *use* the language they have defined. They can see how their language description has been translated into editors and other tools for their language.

By defining a new language, learners effectively create a new tool. In fact, many existing MDE tools define their own language; typically, these are packaged as plugins for IDEs such as Eclipse, IntelliJ, or VSCode. Thus, *learners* temporarily act as *tool providers*.

To hide the details of how MDE tools are integrated into the EP from learners, we allow activities to be coupled dynamically within an activity-configuration file. With this capability, teachers define language-workbench activities by

1. defining an activity for learners to provide relevant descriptions of their new language. A button in this activity triggers a tool function provided by the creator of the language workbench to generate a new MDE tool encompassing the learner’s new language.
2. defining a separate activity that is configured to use the language tool dynamically generated from the learner’s language description.

Listing 5 shows an excerpt from a learning activity where learners produce an Xtext grammar [23] and then try out the generated editor. There are two activities here.

1. activity-xtext defines a panel where the learner can create the grammar. This panel uses the xtext-grammar panel type provided by the Xtext tool, which includes a button to trigger the generation of Xtext artefacts from the grammar. In addition to the usual panel attributes, panel-xtext uses two attributes editorActivity and editorPanel. These refer to the second activity activity-editor and a panel in that activity (panel-editor).
2. activity-editor is the activity learners use to try out their new language. Because the activity is referenced from a panel in the first activity, the EP only makes activity-editor available through the menu if the generation action in the first activity has been used by the learner and has produced an editor. The API endpoint associated with this action is expected to return a URL pointing to where the newly generated tool is available. This URL is made available to the learning activity via the {{ID-panel-editor}} variable, which is used to load the generated tool in the second activity.

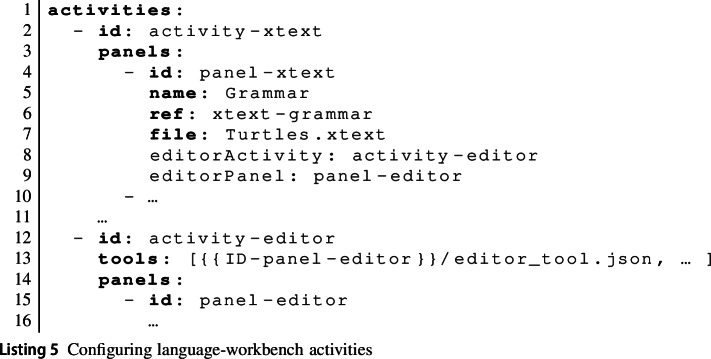


### Contributing MDE tools

Tool services provide the functionality that the installed tools on a developer’s local machine environment would normally provide—for example, model-to-model transformation, text generation, or model validation. They make up the back-end of the EP, providing a wrapper around an existing MDE tool. A tool service comprises a (set of) tool function(s) and static resources.

The tool function provides a web-based API endpoint that conforms to the tool interface specification. The static resources a tool provider must create (**UC7**) include: a tool configuration file, highlighting rules, and icons. Tools are provided independently of learning activities. They may be hosted on the same infrastructure as the EP, but they may also be hosted on separate infrastructure—for example, controlled by the tool provider (R7). Teachers reference tools by their URL to use them in an activity they are creating.

#### Tool configuration

**Fig. 5:**
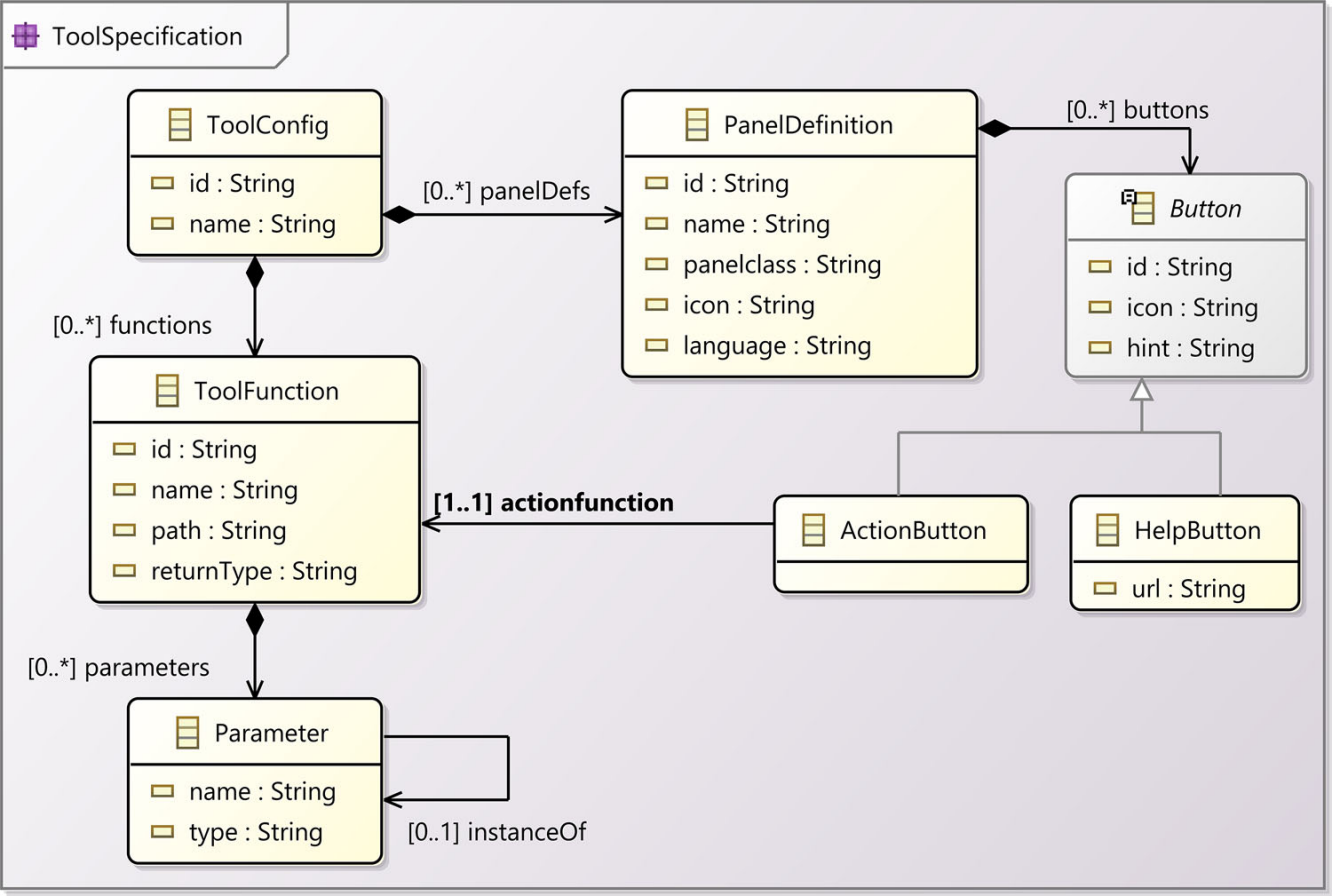
Tool configuration meta-model.

A tool-configuration file defines the tool functions and the panels that are available for a learning activity to use. Figure 5 provides an overview of the concepts used in tool-configuration files.

Listing 6 shows the top-level structure of a tool configuration using the example of the Epsilon tool, which provides access to the various tools in the Epsilon suite [30]. Tool configurations have an id and a name. They then define two key contributions: functions and panelDefs (panel definitions).
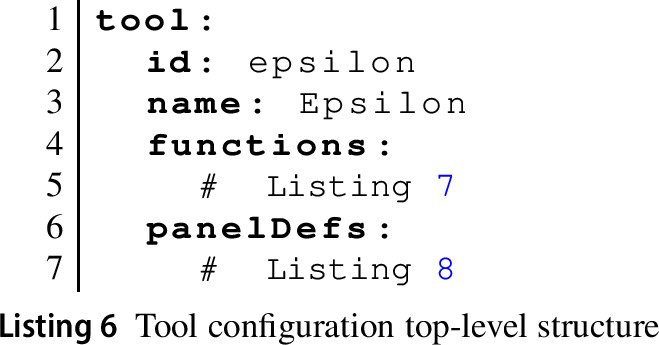


A tool can declare multiple functions. Each function declaration has an id and an explanatory name. The path attribute is a URL that the EP will send a POST request to invoke the function. It is up to the tool service (see below) how to implement this API endpoint. The function declaration further declares the formal parameters of the function and indicates its returnType.

Listing 7 shows the EVL tool configuration function declaration for our example. As can be seen, parameter declarations normally have a name and a type. The latter is used to support a limited degree of automatic type conversion (cf. Section 3.5). The model parameter has an additional instanceOf attribute, indicating that whatever is passed in through this parameter is expected to be a model that is an instance of the meta-model passed in via the parameter named metamodel.
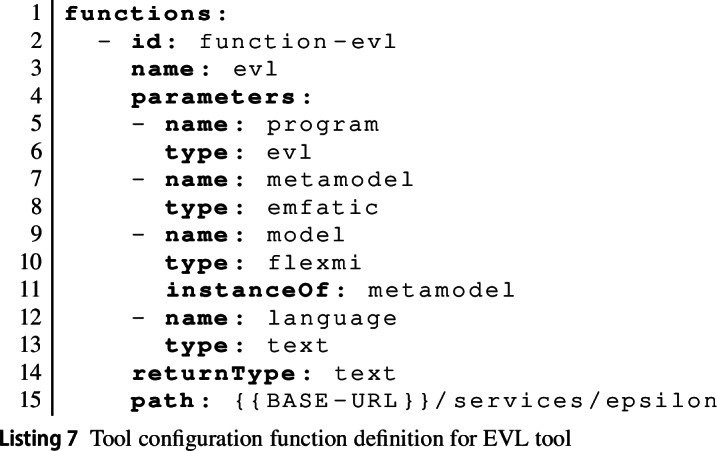


The path uses the special {{BASE-URL}} rewrite rule, which the EP will replace with the location from which the tool configuration file was loaded. This allows tools to be hosted in different locations without the need to change their specifications.

A tool further defines multiple panel definitions (panelDefs) to be instantiated by the panels of activity configurations using the tool. Each panel definition has an id, a name, and an icon (referencing an image provided via a separate CSS file). The panelclass attribute identifies one of a fixed set of base panel types: consoles (ConsolePanel), text editors with syntax highlighting and other IDE functionality (ProgramPanel) based on the ACE editor framework^7^ and configured via a JavaScript module separately provided by the tool service, and output panels that can show code or diagrams (OutputPanel). The language attribute provides a string uniquely identifying the type of the contents of a panel. This will be used together with the type attributes of function parameters to ensure correct input is provided to tool functions. It is also used to select the correct set of highlighting rules from the rules provided separately by the tool. Listing 8 shows the EVL panel definition.
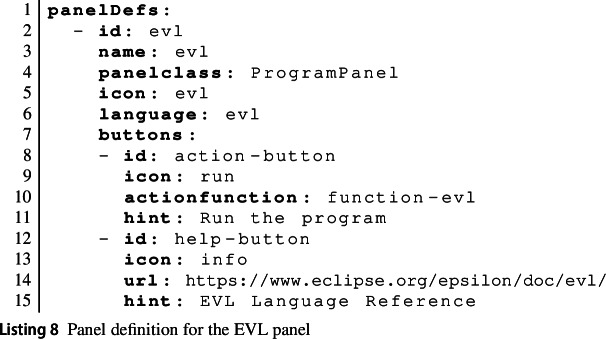


Panel definitions may optionally define an array of buttons. Each button can either be an action button (allowing learners to trigger a specific tool function) or a help button (linking to additional material learners can use to learn more about the contents of that specific panel). Listing 8 shows examples of each type of button.

#### Tool service

A tool’s functionality is provided by a tool service through web API endpoints. These can be implemented in a variety of ways. For many tools, the services will be implemented as stateless functions-as-a-service, but some tools (e.g. language workbenches like Xtext) will use a stateful server as the tool service. Requests and responses use standard JSON encoding for parameters and result data.

### Dynamic invocation of MDE tool functions

As learners progress through learning activities, they edit the contents of panels and click on the buttons available. Action buttons are linked to tool-service functions as described in Sect. 3.4.1. When the learner clicks on such a button, the EP evaluates the linked action definition (cf. Listing 4) to identify the panels whose contents is to be provided as parameter values to the tool-service function.

Tool-service functions expect parameters to be of particular *types*. In particular, models provided are expected to be instances of a particular meta-model and to be presented in a specific concrete syntax.

As different tools are combined in the same learning activity (R6), information will not always be available in the precise type expected by tool-service functions. Rather than requiring tools to provide variants of tool-service functions for a wide range of input types (and type combinations)—which would not scale—the EP provides support for limited implicit type conversion when invoking tool-service functions.

To achieve this, the EP:

1. Includes type information in ToolFunction Parameter specifications to document the type of information expected by the tool-service function.
2. Includes type information in panel definitions (provided by tool configurations) indicating the type of information in a particular panel.
3. Allows tools to register conversion functions from one type to another. These are defined similarly to action functions and are also implemented via a web-based API endpoint.
4. Compares input value types against the expected tool-service function types.
5. Identifies suitable conversion functions to translate provided types to required types and calls them.
6. Calls the requested tool function.

*Types* are represented by strings. Type equality is simply string equality; the EP does not currently provide support for type hierarchies or other advanced features.

The pseudocode in Algorithm 1 shows how the EP handles type conversion when a tool function is triggered by a user clicking on an action button. When an action button is pressed, a $params$ object is created using the corresponding button’s action from the activity-configuration file. The $params$ object maps function names to a value and type. The value is the input to the tool function and is the contents of a displayed panel that is specified by the configuration file’s activity parameter to panel mapping.

**Algorithm 1:**
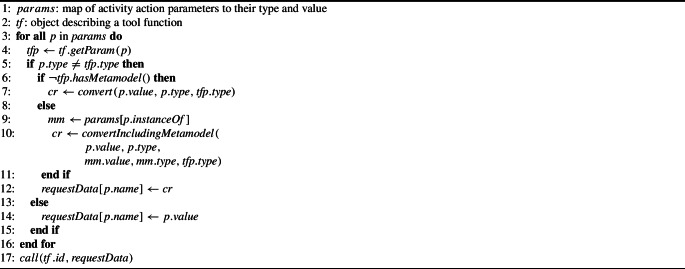
Pseudocode representation of tool-service function parameter type conversion

For each of the parameters in $params$, Line 3, the EP checks to see if the parameter type matches the corresponding tool function’s parameter type, Line 5, to determine whether any type conversion is necessary. If the types match, the value $params$ is inserted into the $requestData$ array on Line 14. If the types do not match, the EP tries to convert the input value to a type that matches the tool function’s parameter type using a conversion function.

To convert a model, the meta-model is required by the conversion function. Converting the format in which meta-models are represented does not require additional reference to an explicit meta-meta-model. The EP handles each of these cases separately. On Line 6 the parameter is checked for a meta-model dependency. If such a dependency exists, this will be captured by the instanceOf element’s presence in the parameter definition (cf. Listing 7), which is extracted on Line 9.

If there is no meta-model dependency, the EP converts the input parameter using the $convert()$ function. If there *is* a dependency, the EP converts the input parameter using the $convertIncludingMetamodel()$ function. This function has five parameters: input value, input type, meta-model value, meta-model type, and target type. Note that $convertIncludingMetamodel()$ may only be able to do the conversion by first converting the meta-model itself to a format that can be accepted by an available conversion function.

The result of either conversion is assigned to $cr$ on Lines 7 or 10, which is inserted into the $requestData$ array on Line 12. Following all the parameters in $params$ being processed, the $requestData$ variable holds the inputs to the tool function with the types it expects. The tool function is finally called by the $call()$ function on Line 17. The $call()$ has two parameters: the id of the tool function, and an array containing the parameters and their values.

Conversion functions are identified from the tool configurations referenced by the activity. To minimise the complexity of the type conversion, only direct conversions using a single function are considered; conversions are not chained. If no suitable direct conversion can be found, the EP reports a configuration error. The EP currently does not perform conversions on tool-service function results, but we plan to introduce this in the future.

### Support available for teachers and tool providers

To support teachers in defining activities (R4) and tool providers in contributing tools to the EP (R7), the abstract syntax of the activity-configuration and tool-configuration languages has been captured in a set of JSON schema definitions [26]. These are used by a VSCode [40] plugin^8^ which provides a degree of validation and code-completion support for teachers creating new learning activities and tool providers contributing new MDE tools. There is also a repository of example activities^9^ that can serve as starting points for teachers to extend and refine, as well as a template repository that can be used as a starting point for new tool definitions.^10^

JSON schemas do not provide support for well-formedness constraints beyond syntactic and multiplicity constraints. When loading a configuration file, the EP provides more detailed validation feedback if required. We plan to translate the current JSON schemas into full DSMLs, which will provide more powerful validation and feedback to teachers and tool providers before loading activities into the EP.

## Case studies

In this section, we present three case studies of different uses of the EP. Each of these demonstrates how different requirements established in Sect. 1 have been addressed by the EP. To structure the case studies, we formulated three exploratory research questions:

1. How can the EP reduce entry barriers for learners and provide a practical, scalable, and supportive learning environment?
2. How effectively does the EP empower educators to design, manage, and control tailored learning activities in MDE education?
3. What mechanisms and processes enable the EP to accommodate contributions from diverse stakeholders, including tool providers, to extend its applicability and utility?

Following the recommendations in [45], the case studies are structured to address these questions by focusing on their real-world context, integrating multiple sources of evidence, and maintaining a clear chain of evidence. Each case study highlights how specific aspects of the platform were used to address one or more of the requirements outlined in Sect. 1, ensuring a thorough and exploratory approach to understanding the EP’s impact and capabilities.

### Epsilon and MDE DevOps

One of the authors (Garcia-Dominguez) presented a tutorial titled “Managing your models as part of a DevOps pipeline” at the 2023 MDENet Annual Symposium.^11^ The tutorial was dedicated to showcasing how model-driven approaches could be combined with DevOps practices: whether by executing model management workflows from continuous integration processes, or by having model management operations support DevOps tasks like interacting with APIs to produce artefacts (e.g. release notes based on the GitHub issues API).

In order to allow attendees (a mixed audience from industry and academia) to interactively try out the examples in the tutorial without having to install and set up an entire development environment, the EP was adopted. This section discusses how workspaces were automatically provisioned for each attendee via the EP and GitHub, and the design and implementation of the various EP activities that were part of the tutorial.

#### Automated provisioning of attendee workspaces via GitHub

The tutorial materials were set up as a GitHub template repository, allowing attendees to launch the EP in different ways depending on their needs (R1) (R3). If the participant did not need to save their changes, they could launch the EP directly on the tutorial materials by following a link in the repository’s README file. This did not require a GitHub account.

On the other hand, if the participant wanted to persist their changes (e.g. to see the automated execution of the CI pipelines after experimenting with the examples), they needed to have their own repository with a copy of the materials. Participants could use existing GitHub facilities to create a repository using the tutorial materials as a template, but they would also need to install the MDENet GitHub application into their GitHub account, so the EP could commit their changes on their behalf.

To avoid this complexity, a GitHub Classroom organisation was created for the tutorial, with the MDENet GitHub application pre-installed into it. A Classroom assignment was created using the template repository as a starting point, and attendees were given an invitation link to have GitHub create a repository within the organisation, which they could use from the EP.

Having created a repository and given the MDENet GitHub application access to it, the next task was telling the EP to open their repository. This required following a link which included the address of the repository. Rather than requiring participants to manually construct the appropriate URL (which would be prone to mistakes), the repository automatically updated its own links in the README by using a GitHub create workflow. Participants only needed to wait briefly for the workflow to complete after creating the Classroom repository, and from then on they only had to follow the updated link.

In general, the attendees did not require assistance with this automated setup (R1), which closely mirrored what would be typically used in an MDE course (**UC1–2**).

#### Model-driven development of Java state machines

The first group of activities that attendees are walked through is the model-driven development of a Java program that implements a state machine. These include:

The above activities reused the tools available from the Epsilon Playground mostly as-is, except for minor changes (R7): adding the JSON metadata needed to describe them as EP tools, and a repackaging as a Micronaut application in order to produce a compact Docker image.^12^

The Epsilon scripts being edited from the activities are exercised in two ways:

- From the EP activities, they are executed independently from each other: while in AV and AT the participant edits the source state machine model and the EVL/ETL scripts, in AG the participant edits the EGL and EGX scripts and an example Java abstract syntax model which is unrelated to the state machine model. The use of a separate Java abstract syntax model is to avoid overwhelming the participant with an overly complex model while learning about EGL and EGX.
- When the participants save their changes, they are committed and pushed by the EP to GitHub, which triggers a CI workflow previously prepared in the repository. The CI workflow uses an existing GitHub action^13^ to run a model management workflow that runs the entire chain of EVL, ETL, and EGL/EGX scripts to generate the final Java code of the state machine, and build it with Gradle.

In combination, this set of activities show how it is possible to showcase various model management languages from the EP and then have the scripts edited by the participants integrate with the existing GitHub CI infrastructure and popular build tools (Gradle) (R3). The use of CI also makes it possible to deliver automated feedback on the edits made by the participants: in an educational setting, this capability could be used to deliver immediate formative feedback.

#### Generation of release notes from GitHub issues API

**Fig. 6:**
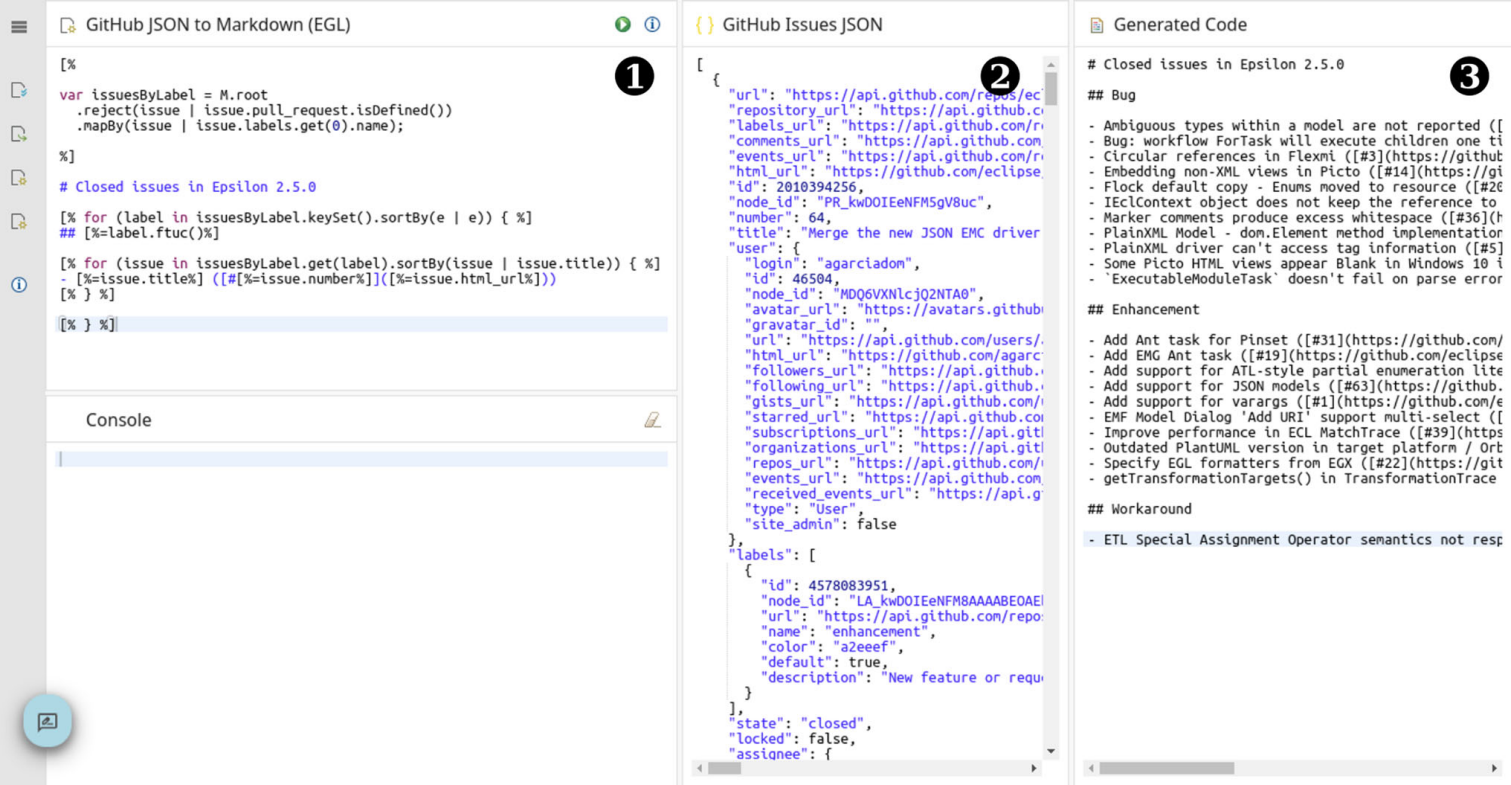
Screenshot of the Github Issues JSON EP activity. Numbered circles indicate different parts referenced from the text.

Besides executing model management operations from CI pipelines, the tutorial included an activity where model management technologies were used to consume information from an existing API, which is more typical of a DevOps environment. The EP activity (shown in Fig. 6) allows participants to experiment with the transformation of the JSON output from the GitHub issues API^14^ to a Markdown document , using an EGL template . Rather than introducing a new model management language, this activity was intended to show attendees that JSON documents could also be used by Epsilon as a model, where its meta-model was implied by the JSON document structure rather than explicitly defined as in EMF models.

Similarly to the AG activity in Sect. 4.1.2, the activity operates from a JSON document in the repository, but the EGL script itself is later used on live responses from the GitHub issues API from a GitHub Actions workflow. This workflow is configured to run periodically, using the existing facilities from GitHub Actions, and upload the resulting Markdown as a build artefact.

#### Transitioning to an IDE

As a last detail, the repository was designed so that participants could at any time transition from the EP to an IDE (R3): in this case, the Eclipse IDE, since the most mature tooling for the Epsilon languages is Eclipse-based. The repository is already a combination of a generic Eclipse project and a Gradle project, which are both directly usable from Eclipse if Epsilon is installed.

This shows an important contrast with the Epsilon Playground: whereas in the Epsilon Playground the examples are entirely self-contained and can be exported in an executable form from their *Download* button, the MDENet EP expects the teacher to have prepared the repository in advance to make it usable from a desktop IDE. While this requires more preparation from the teacher, it also avoids any assumptions from the web-based environment, allowing the teacher to integrate with the IDE and/or build system that better suits their requirements.

#### Analysis of research questions

The case study has provided the following answers to the above research questions:

*RQ1: How can the* EP *reduce entry barriers for learners and provide a practical, scalable, and supportive learning environment?*

Prior to the EP, learners would have needed to download and unpack the starting code for the tutorial, install a recent version of the Eclipse Modelling Tools distribution, set it up with the appropriate plugins from third-party update sites (specifically, Epsilon), import the starting code into their IDE, and resolve any technical issues around their Java environment. The EP simplified this (R1) to one click if they did not want to save their changes (following a link in the README), and only a few more clicks if they did want to save them (creating a repository via GitHub Classroom, waiting for a few seconds, then following a link). An alternative might have been to deliver the learning activities through a prepackaged environment—for example, using Docker. This would have required learners to have the appropriate run-time infrastructure (Docker) available on their machines already, or would have incurred effort for installing such infrastructure. Experience in other learning contexts (see Sect. 4.2) shows that this can be a significant hurdle for some learners.

Likewise, any UI elements that were not needed for the learning experience but which would be part of a full-featured IDE were avoided by using the EP, allowing them to focus on the core topic instead of having to deal with the steep learning curve of a full IDE (R5). Once the learners were comfortable with the concepts, they could transition their work (R3) to the same fully-featured IDE that they would use in a professional setting, by simply cloning their repository and setting it up in the IDE as usual.

*RQ2: How effectively does the* EP *empower educators to design, manage, and control tailored learning activities in MDE education?*

This case study allowed the teacher to cover every stage of a model-driven continuous integration and delivery pipeline (R2)(R4), from the modelling of the state machine to the compilation of the generated code and its packaging as a library. Learners could practice with each stage of the pipeline independently without having to learn the specifics of running each type of Epsilon script, and the CI/CD configuration was already done for them in the starting code (which they would have had to learn to do from scratch otherwise). Teachers were able to easily direct learners’ focus on the appropriate files and actions for each step with minimum effort by providing a declarative specification of the activities.

The compatibility with GitHub Classroom also opens up new opportunities for monitoring the progress of the experience, e.g. by using the reporting tools in GitHub, and regularly inspecting the current state of the various learners’ repositories. Excluding the test repository created by the teacher, there were another 11 repositories created within the GitHub Classroom organisation created for this tutorial. This excludes attendees who simply followed the link in the README without creating their own repository (as they were happy to try out the EP without saving their changes).

*RQ3: What mechanisms and processes enable the* EP *to accommodate contributions from diverse stakeholders, including tool providers, to extend its applicability and utility?*

This tutorial was initially based on the Eclipse Epsilon tool server that was adapted by the EP developers from the first version of the Epsilon Playground, which was based on Google Cloud Functions. The Epsilon developers have contributed a new version of the Epsilon tool server, based on a new framework (Micronaut) with significant improvements in performance and space savings. Since the only requirement is to maintain compatibility with the EP tool API, it has been trivial to swap out the old Epsilon tool server with this new one (R7) without impacting any of the existing teaching materials.

### Developing DSMLs in Xtext

In this section, we describe how the EP was used as part of a course on MDE taught to third-year undergraduate students and master’s students at King’s College London. Through this, we demonstrate how new tools can be easily contributed to the EP (R7) and how they can be combined with existing tools to provide rich learning activities to learners of MDE (R6). The learning activity teaches the use of language workbenches (R2).

We begin by briefly summarising the context in which these activities were introduced, before describing the new Xtext tool service we have implemented. We then show two example learning activities demonstrating the basic use of the Xtext tool service and its combination with existing Epsilon tools.

#### Context: teaching MDE at King’s

At King’s, we teach a course on MDE to third-year undergraduate students and to master’s students. The focus of this course is on developing domain-specific modelling languages (DSMLs), validations, transformations, and other model-management tools. Learning outcomes include understanding core principles of MDE, including constituent elements of language definition in different formalisms, and concepts and technologies for model transformation and code generation, as well as abilities to develop a DSML and support tooling (transformations, code generators, validators). A large part of the practical work in the course uses Xtext [23] to create DSMLs and their supporting infrastructure, but we also use Epsilon tools, and students get some exposure to other approaches for developing DSMLs. The course is taken by between 60 and 100 students each year, comprising both 3rd-year BSc students and MSc students.

A recurring challenge for our students was the installation and use of Eclipse and the various tools required. There is limited TA support to help students resolve technical challenges. Each year, this has caused a significant number of students to struggle to work on the actual learning tasks, because they ran into problems with Eclipse or the tools. In the 2023/24 academic year, we introduced the EP as an optional alternative to reduce the need for students to struggle with Eclipse directly.

#### Xtext tool service: integrating a language workbench

Xtext is a language workbench [22]. This means there are two distinct phases that Xtext learning activities need to be able to support:

1. *language definition,* where learners define (parts of) their DSML using the relevant Xtext concepts
2. *language use,* where learners experiment with their new DSML in an editor with error feedback, code completion, etc., but also by integrating with further tools, such as model transformations.

It is important that learners clearly understand the distinction between these phases, as they will be undertaken by different roles in real-world DSML projects: *language engineers* will define languages and *language users* will use them. We have chosen to represent each phase by separate, but linked, learning activities:

1. In a first activity, learners define aspects of their language. They then select an action button, which sends their definitions to the Xtext tool services, which generates the full set of Xtext artefacts. For each learner, the Xtext tool service generates a tool service providing a panel for editing models in the learner-defined DSML and makes this available via a unique URL.
2. This dynamically generated tool service is then used by learners in a second activity, where they can test their new language.

Note that this requires a *stateful* tool service that can maintain generated Xtext artefacts for each learner. The service implements a single tool-service function that accepts an Xtext grammar and, optionally, a scope provider, a validation implementation, and a code-generator implementation, each using the relevant Xtext interfaces. The tool service function sets up an Xtext project structure and copies in the provided files, before triggering the Xtext generation process. As part of this, Xtext is requested to generate web support,^15^ which is then used as the basis for the dynamically generated tool service. Generated tool services are removed from the server every 24 h to manage memory usage on the server. In the future, we may implement more sophisticated resource management.

The Xtext tool service uses code generation to produce a tool service implementation that is specifically adapted for the language defined by the learners and that contributes the following:

- a panel for editing models in the new DSML, including code completion and error markers
- a conversion function for converting models in the DSML into plain XMI for use with other tools
- an action function for converting models to a diagrammatic representation as an object diagram to allow learners to explore parse results
- an action function for triggering the Xtext code generator.

#### Examples

The first activity learners engage with asks them to create a simple Xtext grammar, generate the language infrastructure, and then experiment with their new language in a simple editor. Similar to the activities presented in Sect. 4.1.2, we distribute learning activities using GitHub Classroom, providing repositories with full Eclipse projects so learners can use them both from the EP and directly from Eclipse (R3). To further support learners, we provide GitHub actions that automatically run tests for every change and allow learners to understand what part of the language they are still missing in their grammar.

**Fig. 7:**
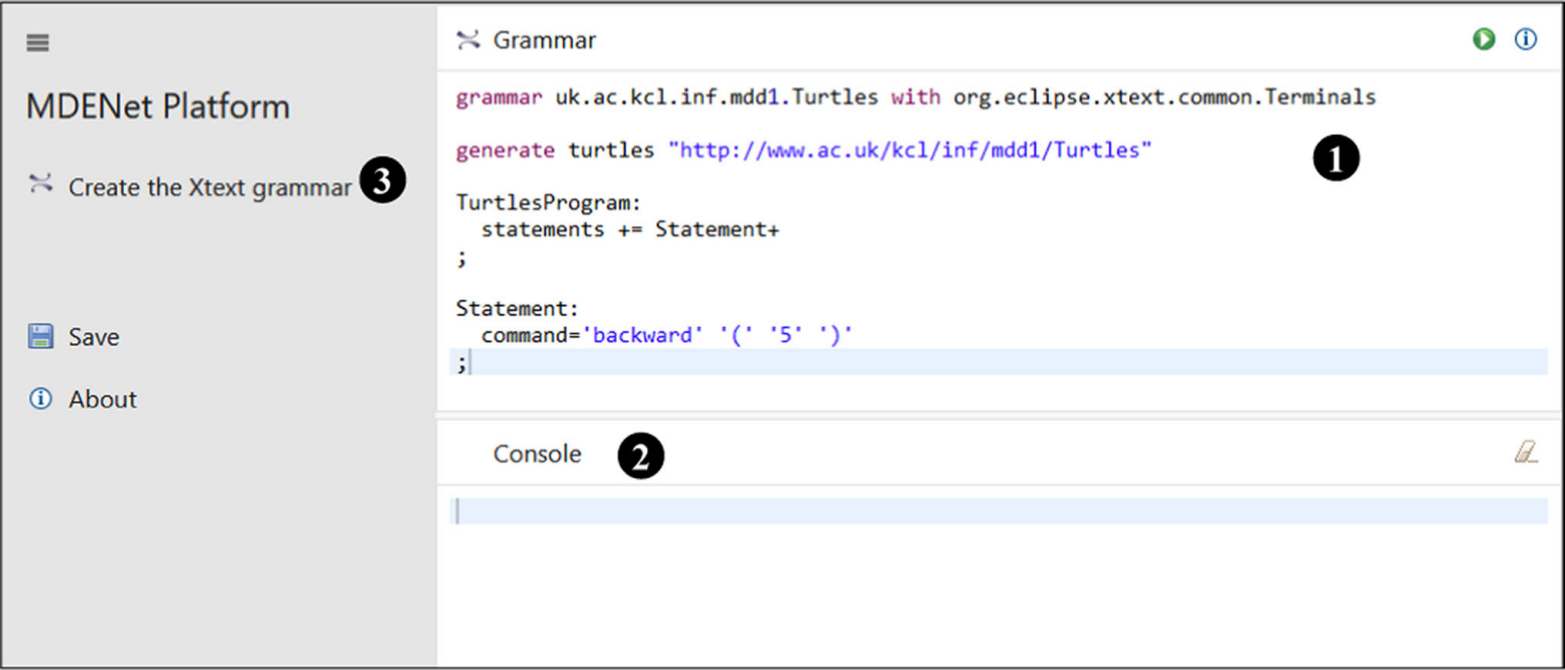
Basic Xtext activity: defining the grammar.

Figure 7 shows the first activity, where learners define a grammar (Panel ) and generate the Xtext artefacts (Button ). As the generation process runs, learners will receive feedback in the console panel ; this is the same feedback they would receive when generating Xtext artefacts in Eclipse and will include error information if there are problems with the grammar defined. Note that the menu  currently only shows one activity; this will be updated as soon as an Xtext editor has been successfully generated from the learner’s grammar.

Listing 9 shows the corresponding activity configuration. This is fairly straightforward (R4), but note:

- the use of the publicly hosted EP (Line 7)
- the reference to a second activity and panel on Lines 14–15, indicating the activity that uses the generated Xtext editor
- the use of the Xtext action function from the action defined on Lines 26–34.

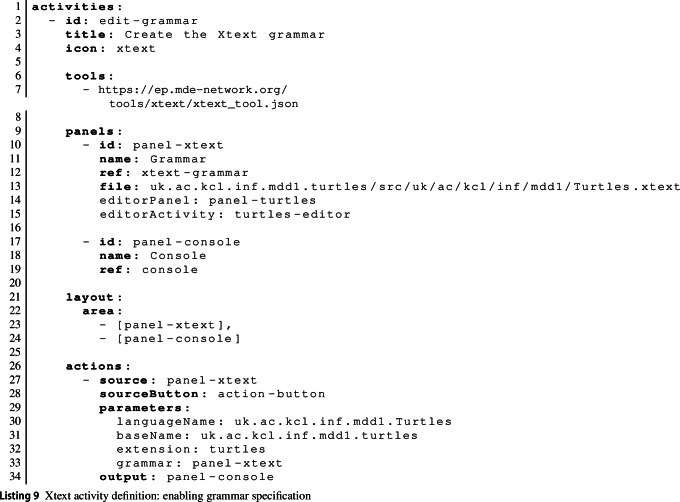


**Fig. 8:**
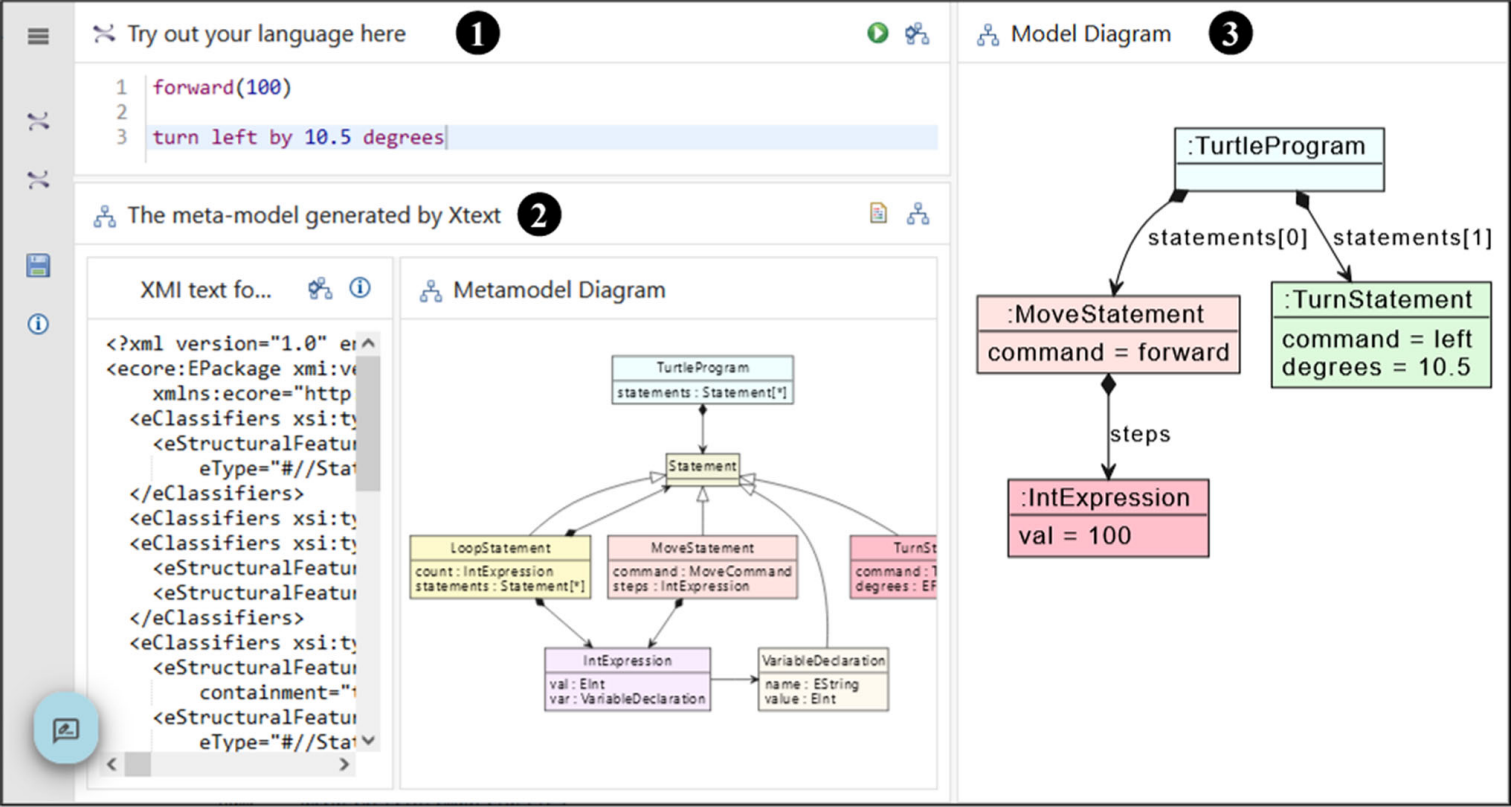
Basic Xtext activity: exploring the language.

Fig. 8 shows a screenshot of the second learning activity, where learners can explore the DSML they have defined. Panel  shows an editor using the syntax highlighting and code completion generated from the grammar. Panel  shows the meta-model generated by Xtext, allowing learners to improve their understanding of Xtext meta-model inference. They can view the meta-model textually (using the Ecore XML rendering) or graphically as a class diagram. When learners click on the  button in Panel , they can see a graphical representation of their current model as an object diagram in Panel . This helps conceptualise the result of parsing the model text into the internal representation used for validation, code generation and model transformation.

Listing 10 shows the definition of this second activity. Note the use of the {{ID-panel-turtles}} rewriting on Line 10 to load the definition of the tool service dynamically generated for the learner. We use the same mechanism again on Line 26 to load the Xtext-generated meta-model. We use the emfgraph panel type provided by the emf_tool to show the model diagrams. This type of panel is able to show an arbitrary SVG diagram. We generate the SVG using an action function (not shown here) that runs an EGL script [43] generating a PlantUML^16^ specification, from which we then generate a SVG.
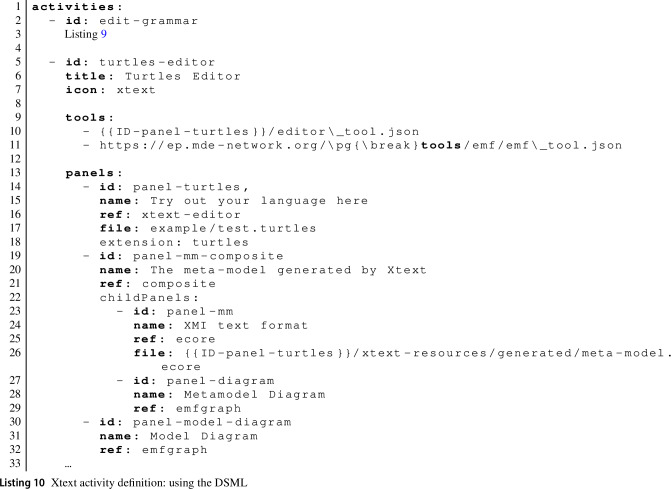


**Fig. 9:**
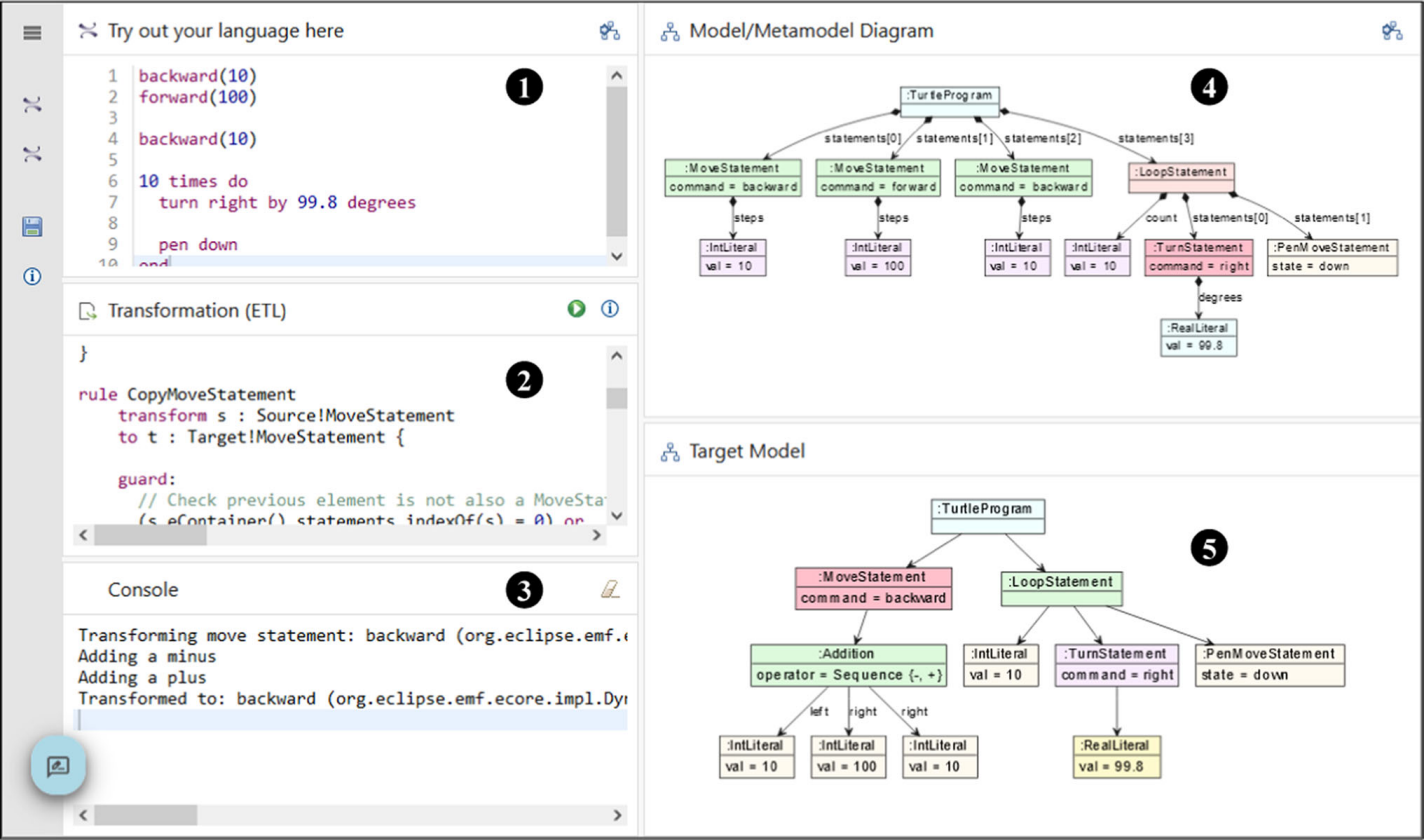
Advanced Xtext activity, combining a learner-defined Xtext editor with an ETL transformation.

Finally, we briefly present in Fig. 9 an advanced activity combining Xtext and ETL [32] (R6). Panel  is the generated Xtext editor, but in Panel  learners are able to define an ETL transformation for models in their newly defined DSML. They can then execute the transformation, which presents a visualisation of the resulting model in Panel  as well as any additional output in the console . Panel  can be used to visualise the current model or the Xtext-generated meta-model. The activity also uses a hidden panel that loads the Xtext-generated meta-model, making it available to be visualised via the button  in the top-right corner of Panel .

Key to the integration of tools in this activity (R6) is the EP’s ability to automatically convert model types. This allows the contents of the Xtext editor panel to be directly provided to the ETL tool, even though the ETL tool does not know the learner-defined DSML. The dynamically generated Xtext tool service provides a conversion function from DSML models to XMI, ETL can accept XMI input, and the EP automatically recognises that it can apply this conversion.

#### Analysis of research questions

The following analysis examines how the Xtext case study demonstrates the EP’s effectiveness in addressing key challenges in MDE education, focusing on learner accessibility, educator empowerment, and stakeholder contributions:

*RQ1: How can the* EP *reduce entry barriers for learners and provide a practical, scalable, and supportive learning environment?* Outside the EP, developing new languages with Xtext introduces significant accidental complexity. Learners must:

(a) install Eclipse and Xtext, ensuring the use of matching versions (b) learn how to generate a new set of Xtext plugin projects in their Eclipse workspace (c) learn how to define an Xtext grammar for their language (d) learn how to trigger the generation process from the Xtext grammar (e) learn how to run a second Eclipse instance with their new language installed (f) learn how to set up a project and file configured for using their new language (g) use the Xtext editor to interact with their new language.

Using the EP, learners only need to do Steps (c) and (g), significantly reducing the entry barriers and allowing learners to focus on MDE concepts rather than the accidental complexity of the tooling (R1). Once learners have understood the key MDE concepts, they are still able to clone the repository underlying the activity and explore it directly in Eclipse (R3).

We asked students for feedback and received three responses—the platform was an optional part of the module and not all students engaged with it at this point—the number of responses is thus too low to allow robust analysis. However, informal student feedback (including via the feedback button on the hosted platform) indicates that students found the platform ‘intuitive’ (e.g. “I liked how easy it was to visualise the meta-model”) and useful. We hope to be able to collect more structured feedback in future instalments of this course.

*RQ2: How effectively does the* EP *empower educators to design, manage, and control tailored learning activities in MDE education?* In this case study, we have shown how teachers can define learning activities (R4) focused on the definition of new languages (R2) – specifically using Xtext. As shown in Fig. 9, teachers are able to combine the definition of new languages with standard model-management activities such as model transformations (R2) by combining a very diverse set of tools such as Xtext and ETL (R6). This would create additional accidental complexity if done directly in Eclipse: learners would need to also learn how to configure ETL to be able to read a model expressed in their new language. The EP removes this complexity and enables teachers to guide learners by limiting what they can do to a single button  for executing the ETL script on the current model without the need for learners to configure the ETL engine first (R5).

*RQ3: What mechanisms and processes enable the* EP *to accommodate contributions from diverse stakeholders, including tool providers, to extend its applicability and utility?* This case study has shown (cf. Sect. 4.2.2) how a new tool (Xtext) can be easily added to the EP (R7) by (a) implementing a simple server wrapping the tool and providing a web API; and (b) describing the tool capabilities in a JSON/YAML tool specification.

### YAMTL playground and analysis with the EP

Yet Another Model Transformation Language (YAMTL) [4, 5] is an expressive model-to-model transformation language that is offered as an internal domain-specific language (DSL) of JVM languages, including Java, Xtend, Groovy and Kotlin. YAMTL is a model-to-model transformation tool available independently of any IDE, where models can be typed with meta-models or can be imported from semi-structured data using flexible models [6]. YAMTL model transformations can be used to define model queries by using pattern matching, out-place model transformations by mapping an input model into a new output model, or in-place model transformations by rewriting a given model.^17^

In this case study, we show how the EP has been instantiated to create an interactive playground for the YAMTL Groovy dialect, which is used within YAMTL’s documentation, for showcasing examples to YAMTL learners (R1). Additionally, we demonstrate how the experience garnered during the definition of the YAMTL playground and documentation highlighted common problems, which we address by deploying a collaborative tool that facilitates the inspection of activity configurations for the EP **(UC5–6)**, showcasing how to analyse YAML/JSON configuration files with activity/tool specifications using YAMTL.

#### YAMTL playground and documentation

An out-place transformation in YAMTL consists of a header declaration specifying the input and output meta-model, rules defining the transformation logic and helpers defining reusable logic across rules. Rules consist of an input object pattern that is used to match a graph of objects in the input model, and an output pattern that determines the graph of objects to be created in the output model. Post-rule operations can also be specified in each rule, defining additional logic at end of the rule application. Helpers can be used to define attribute values, static operations or contextual operations, and their evaluation is cached, speeding up computations.

**Fig. 10:**
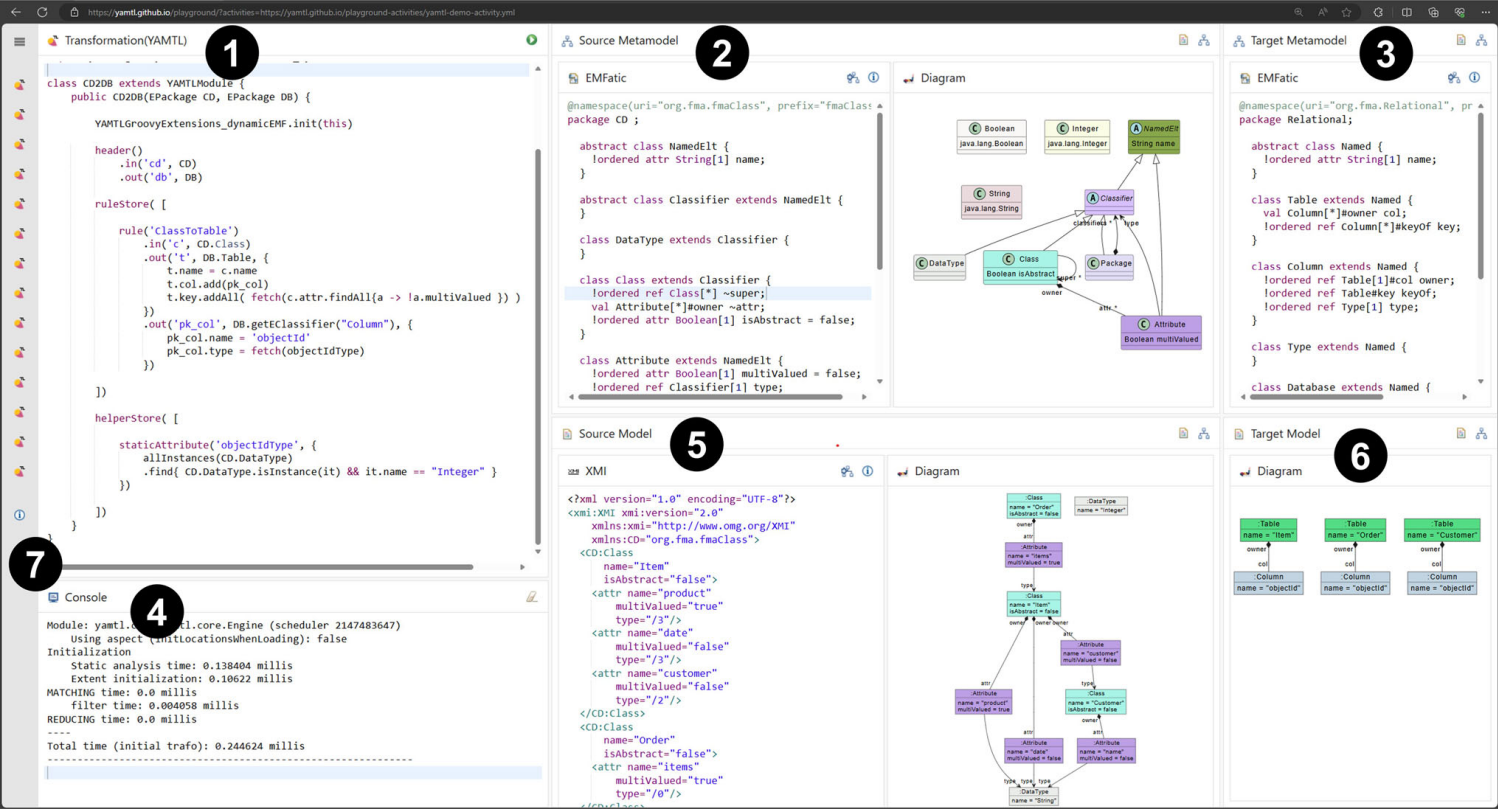
YAMTL Playground.

The YAMTL playground, shown in Fig. 10, enables the development of YAMTL model-to-model transformations using Groovy. The playground displays the model transformation definition  in a Groovy editor, the source meta-model  and the target meta-model  in Emfatic or XMI notation, while the source model  is provided in XMI notation. The execution of the transformation produces an output model  and transformation execution run-time statistics in the output console . All meta-models and models are presented in composite panels that show the models in textual format and can render them in class diagram notation for meta-models and object diagram notation for models. The playground allows for interactive modifications of transformations, meta-models, or models. A number of predefined model transformations are given as examples in the left-hand-side panel .

Although YAMTL does not require any specialised local installation, the EP handles the execution of YAMTL transformations from the browser, thus eliminating the need to use an IDE or configure a Java project (R1). The back-end service for YAMTL has been implemented using an AWS lambda function^18^ for executing the transformation engine (R7). Furthermore, models, meta-models, and transformations are stored in a self-contained Git repository configured as a Gradle project.^19^ This setup makes it easy to reuse the software artefacts and execute them locally using an IDE of choice. Users can seamlessly clone the repository, ensuring that all necessary dependencies and configurations are included, which simplifies the process of running the examples locally and integrating them into other projects or environments (R3). The activity configuration files in the playground refer to the software artefacts contained in those Git repositories using URIs, allowing the configuration of learning activities to be deployed elsewhere, provided that all URIs correspond to accessible resources.

The playground is incorporated in YAMTL’s website as a companion tool for a tutorial to learn the YAMTL language, which contains three types of examples^20^ (R5):

- **Reverse List**^21^: A basic example showcasing the fundamental use of the language for reversing a list. This example helps learners understand the basic syntax and operational semantics of YAMTL, particularly focusing on how to define transformation rules and apply them to simple data structures.
- **Workflow to HTML**^22^: This example is centred around converting flowchart elements into HTML elements, which involves multiple small examples demonstrating a range of YAMTL operations, supporting learners in mastering different aspects of model transformation. Key features covered include:
  - Basic transformation logic: Learners begin by defining simple rules to transform flowchart elements (such as nodes, actions, and transitions) into corresponding HTML elements. This helps in understanding the fundamental syntax and transformation process of YAMTL.
  - Rule inheritance: By implementing rule inheritance, learners can create reusable transformation logic. Abstract rules serve as templates that can be extended by specialised rules, promoting modular and maintainable code.
  - Lazy rules: The example introduces lazy rules, which are executed after all non-lazy rules. This teaches learners about different execution strategies within YAMTL, showing how to use lazy rules for efficiency.
  - Transient rules: These rules perform calculations or updates without persisting their output in the target model, helping learners understand how to manage intermediate transformation steps effectively.
  - Rule filtering and derived elements: Filtering allows specific input objects to be transformed based on conditions, while derived elements enable the use of contextually relevant objects within rules. These features demonstrate how to create precise and context-aware transformations.
  - Multiple sources and targets: The example shows how to handle multiple input objects and produce multiple output objects within a single rule, showcasing the flexibility of YAMTL in complex transformations.
  - End of rule operations: This feature allows for additional operations after the main transformation logic of a given rule, enabling additional side-effects.
  - Rule priority: By setting rule priorities, learners can control the execution order of rules, ensuring that the transformation process follows a specific logical sequence.
  - Helpers: Static attributes, static operations, and contextual operations are introduced as helpers, providing reusable expressions and methods that simplify the transformation logic.
  Each feature is illustrated with practical examples, ensuring that learners can see how theoretical concepts are applied in real-world scenarios. This detailed walkthrough not only enhances their understanding of YAMTL’s capabilities but also equips them with the skills to tackle complex transformation tasks independently.
- **Fill-in-the-Gap Examples**^23^: These examples allow learners to practice the use of the language at different levels of complexity. Tasks include:
  - Creating an additional type of object in an output model within a rule. This teaches learners how to extend transformation logic to produce new types of objects, enhancing their understanding of output pattern specification.
  - Specifying conditional application of rules. This helps learners understand how to control the execution of transformation rules based on specific conditions, emphasising the importance of guards in rule application.
  - Rule inheritance: This demonstrates how to reuse and extend existing transformation logic by inheriting rules, promoting efficient rule management and modular design.
  - Defining several output elements in a rule to create a complex graph of objects in the output pattern. This example shows learners how to construct complex output models from simpler input models, reinforcing their skills in pattern matching and object graph construction.
  - Using lazy and non-lazy rules. This distinction is crucial for learners to grasp the execution strategy of YAMTL transformations, teaching them when and how to use each type of rule for optimal performance.
  - Resolving object references: This example focuses on managing references between objects in the input and output models, a key aspect of maintaining model consistency during transformation.
  - Using helpers. This emphasises the use of helper operations to encapsulate reusable logic, promoting code modularity and maintainability.

Each of these examples is available on the interactive playground and is accompanied by a solution and is available as a Gradle project, making it easier for learners to validate their work and understand the correct application of YAMTL concepts in practical scenarios.

While developing the YAMTL playground and documentation, we encountered issues with activity configurations becoming lengthy and challenging to debug. This motivated the development of a new tool within the EP to inspect and analyse these configurations more efficiently, as explained in the following subsection.

#### Analysis of activity and tool specifications

In the EP, activities are defined using YAML or JSON configuration files that specify the layout of the front-end and the examples used in the activities. Analysing these specifications is crucial for ensuring that activities are correctly configured and function as intended, especially when dealing with complex configurations. This analysis helps identify and resolve errors early, improving the overall reliability and effectiveness of the configuration of learning activities.

During the development of the YAMTL playground, we observed that these configuration files can become lengthy and challenging to debug because they lack type discipline−meaning they do not enforce strict types−and specify references by name. This is further complicated when multiple activities are included in the same configuration file, displayed in the left-hand-side panel of the EP. Therefore, thorough analysis and debugging of these files are essential to maintain the integrity and functionality of the learning activities.

To address these challenges, we used YAMTL to import activity configurations, available as YAML or JSON files, as flexible models [6]. These are then transformed into models of the meta-model presented in Figs. 4 and 5, so that they can be visualised and analysed. YAMTL model queries^24^ are used to inspect them, aiding in both understanding and debugging. By leveraging YAMTL’s support for flexible models, model transformations, and model queries, users can efficiently examine and troubleshoot their learning activities, ensuring they adhere to the intended structure and behaviour ((R6) & (R7)). This tool has been configured as an EP activity itself,^25^ making the inspection logic accessible for any user to validate (and debug) new learning activities by providing the specification of their activities and tools ((R2) & (R4)), as explained in sections 3.3 and 3.4, respectively.

**Fig. 11:**
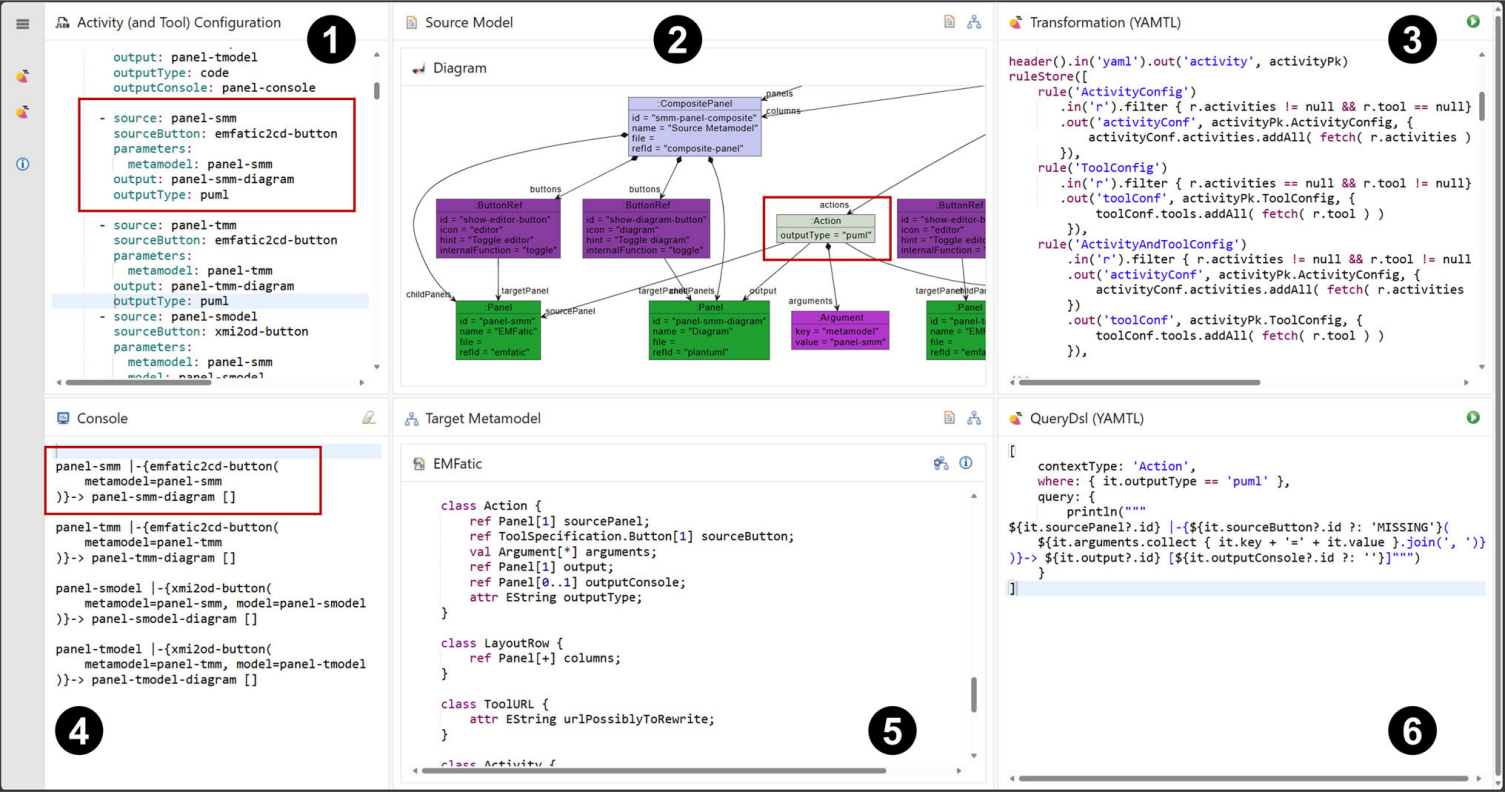
YAMTL inspection of activity configurations for the EP.

The activity, shown in Fig. 11, uses the YAML configuration file  from the activity in Fig. 10 used to configure the YAMTL playground in the previous subsection. This configuration file may include the specifications of activities and, optionally, the specifications of tools. The YAMTL model transformation  converts the YAML/JSON file  into a model  that conforms to the EP meta-models, transforming references by name in the YAML configuration file to references by value in the model. The benefit of this transformation is that models can then be visualised as object graphs using object diagram notation in the EP, as shown in the composite panel , facilitating inspection and analysis of learning activities as explained next.

This activity uses another model query tool  for defining object-oriented queries over models built atop YAMTL pattern matching facilities.^26^ Queries are defined as records, resembling JSON documents, with the following fields: a context type from the meta-model ; a where Groovy closure that specifies which objects from the model  are affected by the query; and a query Groovy closure that traverses the model from an instance of the context type, printing the desired information in the output stream, which is displayed on the console . If, in addition to activity specifications, tool specifications are provided in , users can perform full analysis over the complete specification of an activity, which includes references to tool services.

The tool has been integrated by using an AWS lambda function that takes a meta-model, a model and a query to perform the query. The output stream is captured while the query is evaluated and returned as output so that it can be displayed in the console . In the lambda function, the context type and the where clause are used to define a pattern in a YAMTL rule with a single input element, while the query closure is used as a post-rule operation^27^ (R7).

The query in the example finds out how the activity actions prompt UI state changes, by listing the source-panel containing the action, the button linked to the action, the argument binding for the parameters of the MDE tool linked to the action, and the target-panel containing the results of the tool and any additional output side effects, using the format $\texttt {source-panel}$ $\texttt {|-\{button(parameter-}$ $\texttt {binding)\}-> target-panel [output]}$. In this query, when the button identifier cannot be resolved, MISSING is diplayed to report an error. For the example provided in Fig. 11, the highlighed action in  is imported as an instance of the Action class of the meta-model, shown in , in , via the transformation in . The query  is then used to validate that the sourceButton associated with the action could be resolved to a button in a tool in  as it displays the name of the button emfatic2cd-button and not MISSING. Note that the query is obtaning the name of the button using the expression it.sourceButton.id where it is an Action, which requires the reference Action.sourceButton to have been resolved correctly.

The use of the console and query DSL provides several benefits:

- Improved debugging capabilities: The console allows users to see real-time feedback on their queries, including error messages and execution results. This immediate feedback helps users quickly identify and correct issues in the configuration of their learning activities. The query DSL simplifies the process of writing and understanding complex queries, making it easier to trace the flow of data and logic, and thereby pinpoint and resolve errors.
- Enhanced interactivity: The interactive nature of the playground enables users to experiment with different analysis queries on-the-fly. This promotes a deeper understanding of the underlying configuration models by allowing users to iteratively define complex model queries over the configuration of their learning activities and find errors more easily.
- Efficiency in analysis and validation: By using the query DSL, users can create reusable query definitions that can be easily modified or extended for different analysis tasks. The console’s ability to display query results in a structured format helps users visualise the impact of their queries, ensuring that the output conforms to the expected format.

#### Analysis of research questions

The following analysis examines how the YAMTL case study demonstrates the EP’s effectiveness in addressing key challenges in MDE education, focusing on learner accessibility, educator empowerment, and stakeholder contributions:

*RQ1: How can the* EP *reduce entry barriers for learners and provide a practical, scalable, and supportive learning environment?* The YAMTL playground, as described in Sect. 4.3.1, eliminates installation and configuration barriers by enabling the execution of model transformations directly in the browser (R1). This design provides immediate access to MDE activities, allowing learners to focus on core tasks without technical overhead. The playground also offers self-contained examples that can be downloaded and executed locally, ensuring a seamless transition to real-world environments (R3). Furthermore, the examples and fill-in-the-gap exercises presented in the playground, as detailed in Sect. 4.3.2, help scaffold learning, guiding learners in experimenting with the language ((R4) & (R5)).

*RQ2: How effectively does the* EP *empower educators to design, manage, and control tailored learning activities in MDE education?* Prior to the EP, model management solutions, such as model validation, were explained conceptually in lectures and their implementation was demonstrated in lab sessions using Groovy programs with internal DSLs. Preparing learning resources involved explaining where to retrieve the code, how to import the project, where each software artefact could be found, how additional tools (like EMF) worked, and then presenting the exercise. The EP simplifies the process by providing readily available examples that illustrate the concepts from lectures, offering two key benefits (R5): *a)* an *online* playground where learning activities can be configured and accessed, and *b)* a significant reduction in cognitive load, allowing educators to focus on the core concepts discussed in lectures, thus bridging the gap between theory and practice. In particular, educators benefit from the flexible configuration mechanisms provided by the EP, as discussed in Sect. 3.4.

*RQ3: What mechanisms and processes enable the* EP *to accommodate contributions from diverse stakeholders, including tool providers, to extend its applicability and utility?* This case study demonstrates how the platform can be extended with additional functionality. Section 4.3.1 illustrates how to integrate the YAMTL model-to-model transformation language, available as an internal DSL in the Java ecosystem (with Groovy used in the examples), where the back-end transformation engine is deployed on AWS using a serverless model. This functionality is particularly useful for a teacher who illustrates MDE examples using YAMTL (R5) and enables other stakeholders to reuse YAMTL learning activities and tools within the EP. Section 4.3.2 explains how the EP can be extended to enhance the configuration and debugging of MDE learning activities. JSON configurations are transformed into meta-model-based models using YAMTL, enabling model analysis for collaborative debugging of activities (R4) using a query language built on YAMTL, as well as model visualisation with third-party tools (R6), an EP tool that renders EMF meta-models and models as PlantUML diagrams (class diagrams and object diagrams, resp.). The model analysis use case also allows tool providers to collaboratively debug activity configurations (R7), while helping them become more familiar with the EP’s activity configuration language through the use of queries to navigate and analyse specific activities (R2).

## Related work

To the best of our knowledge, no other generic playground solution for MDE exists. Playgrounds for specific tools do exist. For example, the Epsilon Playground [33] enables web-based use of the various tools and languages in Epsilon [30]. Its architecture makes use of Functions-as-a-Service (FaaS) for its back-end functions, allowing on-demand scalability and minimal running costs when the platform is not being used.

As discussed in Sect. 3, the EP has been inspired by the Epsilon Playground; however, it has a more elaborate and flexible architecture to allow for declarative description of learning activities (which are hard-coded in the Epsilon Playground) and for integration of a wider range of MDE tools (the Epsilon Playground only supports languages of the Epsilon platform).

Langium [44] also provides a bespoke playground service for basic language-workbench functionalities. A web-based platform for the MontiCore language workbench [34] based on JupyterLab [13] has been used for teaching the tutorials of a conference and lectures on the use and engineering of Domain Specific Languages (DSL).

The relative scarcity of web-based MDE playgrounds can be attributed to the niche adoption of MDE technologies, as well as to the fact that most open-source model management technologies (e.g. ATL, Acceleo, Xtext, Xpand) are implemented in Java. In the absence of a fully-featured, freely-available and performant solution for transforming Java source code or bytecode into JavaScript or WebAssembly, running such a playground requires a client–server architecture. This approach, as demonstrated by the MDENet Education Platform, incurs ongoing operational costs, which can be an additional barrier.

In addition to web-based playgrounds, there are web-based versions of IDEs such as Eclipse [18, 19] and Visual Studio Code [40]. Some code repositories use such online IDEs to provide direct access to repositories, including in educational settings. For example, GitHub Classroom offers access to CodeSpace IDEs (based on VSCode) for learners undertaking activities provided through GitHub repositories [24].

Online MDE platforms have seen increasing interest recently—examples include AToMPM [46], Freon [48], and Gentleman [36]—though note that these tools have not been developed specifically for educational purposes. Umple [37] is an online modelling platform, focused on UML-style models and code generation from them. It is education-focused, but only provides support for a fixed set of modelling languages and tools.

There is a lack of commercial tools available that have an educational focus or offer easy installation and activity-configuration options. We are aware that the desktop-based commercial MetaEdit+ language workbench can be accessed through a browser using a remote desktop service and integrated with open-source model management tools [21]. This probably comes closest to a no-install option that could be used for teaching. However, its significant licensing costs have so far prevented any of the authors from using it for educational purposes.

Finally, PapyGame [9] is a Papyrus-based tool for gamifying software modelling in an educational context. PapyGame currently is desktop-based but the authors envision a web-based version in the future to address installation and configuration challenges.

There are no widespread web-based model formats for online editing and interchange of models that can be used to help integrate different tools of an education platform. An early initiative includes LIonWeb [47]; however, it is in the specification stage.

## Conclusions and outlook

We have presented the MDENet Education Platform (EP), an online playground platform for teaching model-driven engineering. This allows learners to engage with MDE learning activities without having to install tools (R1), including activities that require the definition of new modelling languages (R2). Learners are able to access the activities via the browser, but also via standard tools (R3), as long as the teacher has provided a suitably structured repository with the activity. Teachers define new activities via a declarative specification in a GitHub repository (R4). The specification gives them full control over what the learner can see and do (R5), so that teachers can reduce the accidental complexity learners have to overcome. Teachers are able to combine several different modelling tools into one learning activity (R6). New tools can be contributed to the platform with relatively little effort by wrapping them in a web-based API and providing a declarative description of the functionality available (R7).

We have demonstrated the capabilities of the EP in three case studies, which showcase how different sets of requirements are addressed by the platform. Table 1 summarises which requirements are demonstrated in which case study.

**Table 1:** Mapping of requirements to case studies demonstrating how they are addressed by the EP. $\checkmark$ symbols in parentheses indicate partial demonstration of a requirement. A detailed discussion is provided as a subsection in each case-study description

Requirement	Case Study 1	Case Study 2	Case Study 3
(R1)	$\checkmark$	$\checkmark$	$\checkmark$
(R2)	($\checkmark$)	$\checkmark$	($\checkmark$)
(R3)	($\checkmark$)	($\checkmark$)	$\checkmark$
(R4)	$\checkmark$	$\checkmark$	$\checkmark$
(R5)	$\checkmark$	$\checkmark$	$\checkmark$
(R6)		$\checkmark$	$\checkmark$
(R7)	$\checkmark$	$\checkmark$	$\checkmark$

***Future work.***

An important focus of our work is to make it even easier for teachers to define learning activities. To this end, we are working towards a DSML for activity specification, which will provide improved capabilities for consistency checking as well as for discovering capabilities while building activities.

We are also interested in empirical evaluation of the benefits of the platform in diverse teaching contexts. We will continue to develop the platform and use it in our teaching of modelling and MDE. This will provide opportunities for obtaining more informal feedback from our students. We will also organise formal evaluation experiments, which compare learners using Eclipse and the EP for a range of learning activities.

At the moment, the EP does not yet support graphical modelling languages. We plan to add capacity for such languages in the future, as well as support for Language Server Protocol (LSP).^28^ This will also help improve the validation support for existing MDE tools. We are also exploring opportunities for learners to engage with activities through other user interfaces, most importantly Visual Studio Code, which would enable more seamless integration with GitHub Classroom. The platform’s education focus also creates opportunities to experiment with more powerful and interactive ways of providing feedback on learners’ attempts at completing MDE assignments. Finally, we are working towards integrating the EP with learning-pathway tools—like [10, 11]—so that it can more fully serve as a platform for Open Educational Resources (OERs) [42] in MDE.

***Getting involved.***

This is still a relatively new project, and we encourage the community to get involved. We are very interested in learning from, and collaborating with, others who are trying the platform in their own teaching. If you want to get involved, check out the platform on GitHub^29^ and get in touch with the authors. A publicly hosted version of the platform is available^30^ free of charge for reasonable use.

## Footnotes

1 https://classroom.github.com.

2 This primarily required creating declarative specifications of the activities and the Epsilon tools in the format required by the EP

3 https://github.com/mdenet/educationplatform-docker.

4 https://tinyurl.com/epEpsilon.

5 https://docs.github.com/en/apps.

6 Note this is an approximation of the JSON schema adjusted for readability as a meta-model. A transformation creating instances of the meta-model from JSON files will be presented in Sect. 4.3.2

7 https://ace.c9.io/

8 https://github.com/mdenet/educationplatform-vscode/

9 https://github.com/mdenet/educationplatform-examples/

10 https://github.com/mdenet/educationplatform-tooltemplate

11 Materials available from: https://github.com/agarciadom/mdenet-mde-ci-tutorial

12 The Micronaut application with the Epsilon tools for the EP is available from: https://github.com/epsilonlabs/playground-micronaut.

13 The GitHub action used to run Epsilon workflows is available from: https://github.com/committed-consulting/epsilon-ci-action

14 https://docs.github.com/en/rest/issues?apiVersion=2022-11-28

15 https://eclipse.dev/Xtext/documentation/330_web_support.html

16 https://plantuml.com/

17 See the language reference at https://yamtl.github.io for further information.

18 https://github.com/yamtl/yamtl-playground-backend-tool

19 https://github.com/yamtl/examples

20 Borrowed from https://mde-docs.github.io/.

21 https://yamtl.github.io/examples/linked-list-reversal-example.html

22 https://yamtl.github.io/examples/flowchart-to-html-example.html

23 https://yamtl.github.io/exercises/flowchart-to-html-worksheet.html

24 https://yamtl.github.io/examples/query-dsl.html

25 https://yamtl.github.io/examples/ep-inspection.html

26 https://yamtl.github.io/examples/query-dsl.html

27 For further information, see: https://yamtl.github.io/examples/query-dsl.html

28 https://microsoft.github.io/language-server-protocol/

29 https://github.com/mdenet/educationplatform/

30 https://ep.mde-network.org/
